# Standardization and learning curve in laparoscopic hernia repair: experience of a high-volume center

**DOI:** 10.1186/s12893-023-02119-y

**Published:** 2023-07-28

**Authors:** Francesco Brucchi, Federica Ferraina, Emilia Masci, Davide Ferrara, Luca Bottero, Giuseppe G. Faillace

**Affiliations:** 1 Department of General Surgery, Sesto San Giovanni Hospital, Viale Matteotti, 83, Milan, MI 20099 Italy; 2 Department of General Surgery, Edoardo Bassini Hospital, Cinisello Balsamo, MI Italy

**Keywords:** Inguinal hernia, TAPP, Transabdominal preperitoneal, Critical view of safety, Mesh, Groin hernia, Laparoscopy

## Abstract

**Purpose:**

Groin hernias are a common condition that can be treated with various surgical techniques, including open surgery and laparoscopic approaches. Laparoscopic surgery has several advantages but its use is limited due to the complexity of the posterior inguinal region and the need for advanced laparoscopic skills. This paper presents a standardized and systematic approach to trans-abdominal pre-peritoneal (TAPP) groin hernioplasty, which is useful for training young surgeons.

**Methods:**

The paper provides a detailed, step-by-step description of the TAPP based on evidence from literature, anatomical knowledge, and the authors’ experience spanning over 30 years. The sample includes 487 hernia repair procedures, with 319 surgeries performed by experienced surgeons and 168 surgeries performed by young surgeons in training. The authors performed a descriptive analysis of their data to provide an overview of the volume of laparoscopic hernioplasty performed.

**Results:**

The analysis of the data shows a low complication rate of 0.41% (2/487) and a low recurrence rate of 0.41% (2/487). The median duration of the surgery was 55 min, while the median operation time for surgeons in training was 93 min, specifically 83 min for unilateral hernia and 115 min for bilateral hernia.

**Conclusions:**

The TAPP procedure appears, to date, comparable to the open inguinal approach in terms of recurrence, postoperative pain and speed of postoperative recovery. In this paper, the authors challenge the belief that TAPP is not suitable for surgeons in training. They advocate for a training pathway that involves gradually building surgical skills and expertise. This approach requires approximately 100 procedures to achieve proficiency.

## Introduction

Groin hernias are a common condition that affects millions of people worldwide [1]. The occurrence is 27–43% in men and 3–6% in women [2]. The majority of cases are symptomatic, but even those that present without symptoms have a high chance (70%) of becoming symptomatic within 5 years [3].

The most frequent surgical technique for repairing groin hernias is the Lichtenstein repair, but more advancements in laparoscopic surgery have led, over thirty years ago, to the development of techniques such as the laparoscopic transabdominal preperitoneal (TAPP) and totally extraperitoneal (TEP) repair [4], [5].

Laparoscopic surgery is associated with several benefits over traditional open surgery, such as a lower risk of wound-related complications, a faster return to work and activities, and less chronic pain [6]. Nevertheless, certain research also reports higher incidences of seroma formation and relapse with laparoscopic inguinal hernia repair [4], [7], [8].

Despite the fact that this laparoscopic approach is well-established and brings numerous benefits, the popularity of this technique is not yet at its peak [1], [7].

The reason why this minimally invasive technique is struggling to gain acceptance may be explained by the following: the possibility of causing significant harm during a procedure that is typically considered a minor routine surgery for a benign pathology if performed laparotomically, can be a discouraging factor for adopting this technique [8]; it is necessary to learn and become familiar with a new anatomy, that of the posterior inguinal region; advanced laparoscopic skills are required due to the fine movements that need to be performed and due to the uncomfortable working position. In fact, many surgeons who have approached this technique have started from a robotic approach, which allows for easier fine movements and makes it easier to learn this procedure [6], [7]. We believe that, regardless of the surgical technique used, it is essential to start from anatomy and its systematic approach.

The operative principles of the TAPP technique were described in 1992 by the French surgeon Maurice Arregui, which involves dissection of the pelvic floor, placement of a large and flat prosthesis, and avoiding an onlay prosthesis placement [4].

The standardization of the anatomical landmarks of the posterior inguinal region has been greatly contributed to by the work of Daes and Felix in 2017 [9]. They applied the concept of the critical view of safety, used in laparoscopic cholecystectomy [10], to the laparoscopic repair of inguinal hernias, coining the term “critical view of the myopectineal orifice (MPO)”. The Critical View (CV) of the MPO is achieved through a set of steps that ensure proper exposure of the anatomical area before placement of the mesh. It defines the standard that must be met in order to achieve successful surgical outcomes [9].

Furthermore, Furtado et al., in 2019, built upon this work by introducing the theory of the inverted Y and the 5 triangles, which greatly helped those looking to begin performing this surgical technique. This image is formed by the placement of the inferior epigastric vessels superiorly, the vas deferens medially, and the spermatic vessels laterally. By locating the iliopubic tract, which runs horizontally through the deep inguinal ring at the center of the inverted Y, five distinct areas can be visualized, which are referred to as “Five Triangles” in a didactic manner [11]. Subsequently, Claus et al. have divided the area to be dissected into 3 zones and have established the 10 golden rules for performing a laparoscopic inguinal repair. In summary, three zones established are: Zone 1: corresponds to the lateral area to deep inguinal ring and spermatic vessels; Zone 2: is medial to inferior epigastric vessels and vas deferens and corresponds to the site of direct hernias; Zone 3: represents the operative area that demands more attention which includes inferior epigastric vessel and deep inguinal ring superiorly and spermatic cord elements and external iliac vessels [5].

This article stems from our team’s extensive experience in laparoscopic transperitoneal repair of inguinal hernias. We began performing this type of surgery in 1992 and, to date, have completed over 3500 procedures. Over the years, we have standardized our surgical technique and made it easily replicable by surgeons in training.

Herein, we describe a step-by-step useful approach with a review of some concepts related to the standardization of the anatomical landmarks of the posterior inguinal region. The aim of this article is to try to standardize as much as possible and give some tips to surgeons who want to approach this technique for the first time.

## Methods

The description step by step of the presented surgical technique derives from the mixture of theoretical knowledge, scientific evidence and direct experience of a center with over twenty years of experience in laparoscopic abdominal wall surgery.

The technique has been developed and perfected by experienced laparoscopic surgeons.

The surgeries took place in two different hospitals in the period between March 2015 and October 2022. Most of them were performed by experienced surgeons (319) and the others (168) by younger surgeons in training. Different information was collected: personal data (age and sex), characteristics of the hernia treated (position, possible recurrence, operating setting) and data relating to the surgery (operation time, type of mesh used, closure technique of the hernia defect). The data collection took place via CRFs on an excel sheet shared between different project managers. Follow-up took place via telephone call, with simple and direct questions about the presence of chronic pain and possible recurrence of groin swelling, ability to carry out normal daily activities in the absence of complaints, ability to perform greater activities than daily activities in the absence of complaints. Patients who reported symptoms were called in for an outpatient evaluation.

### Surgical technique

**Fig. 1:**
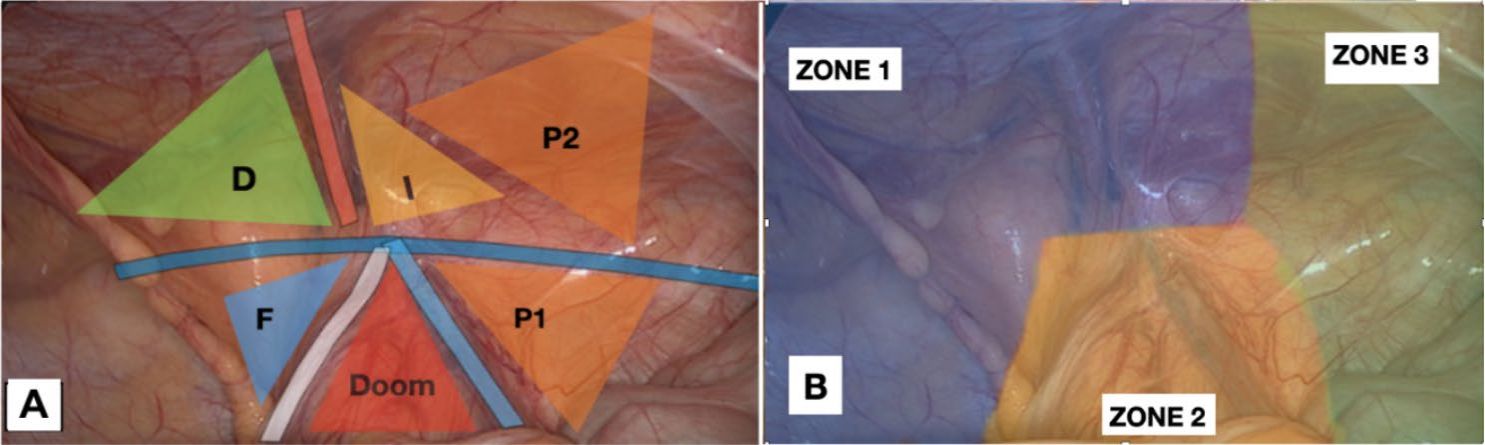
(**a**): Revised anatomical landmarks: P2 stands for the second triangle of pain. It covers the varying anatomy of nerve branches and the transversus abdominis muscle area. (**b**): Revised division and numbering of the posterior inguinal region

During our experience, we came to consider the division of the anatomical area into zones and triangles (Fig. 1a and b), also reported by Furtado et al. and Claus et al., as fundamental for standardization and replicability of the procedure. However, we recommend a different numbering: zone 2 of Furtado et al. becomes zone 1, which is also reviewed in its extent. Starting from the peritoneal incision, the dissection is carried out near the first anatomical landmark (epigastric vessels) above the internal inguinal ring. In our opinion this area could be included in zone 1. Then, the dissection is moved medially into zone 1 to look for the second anatomical landmark: the Cooper’s ligament. If a direct inguinal hernia is present, it will be necessary to reduce the hernial sac before visualizing the Cooper’s ligament.

After correctly visualizing the two landmarks in zone 1, the surgeon moves into Zone 2 (zone 3 according to Furtado et al.) and proceeds with the reduction of any indirect hernia sacs and the parietalization of the vas deferens (anatomical landmark 3 A) and the identification of the spermatic vessels (anatomical landmark 3B). Finally, zone 3 (zone 1 according to Furtado et al.) is prepared with attention to the two pain triangles (P1 and P2) until sufficient space is available for the prosthesis (Fig. 1b) [11].

We have noticed that, in particular, young surgeons at the beginning of their experience with TAPP, had less difficulty starting from zone 2 of Furtado, which we consider the starting point of the dissection (zone 1). Beginning with this area and clearly visualizing the Cooper’s ligament allows for better and safer following of the anatomical planes in the subsequent zones.

The surgical technique could be standardized and divided in phases as follows:

1. *Placement of the trocars* (Fig. 2): The positioning of trocars is crucial in laparoscopic inguinal hernia repair. If placed incorrectly, the procedure can become technically challenging. The principle of positioning is always based on triangulation. The optical trocar is always supra-umbilical. In unilateral hernias (Fig. 2a), the trocar on the opposite side (5 mm) as the defect should be placed in a point that is one-third lateral of the distance from the anterior superior iliac spine to the umbilicus (such as a bilateral Mc Burney’s point). The trocar on the same side (10 mm) is located at the height of the transverse umbilical line, halfway between the anterior axillary line and the midclavicular line. The ipsilateral trocar is positioned higher for increased ease in opening and closing the peritoneum.
  **Fig. 2:**
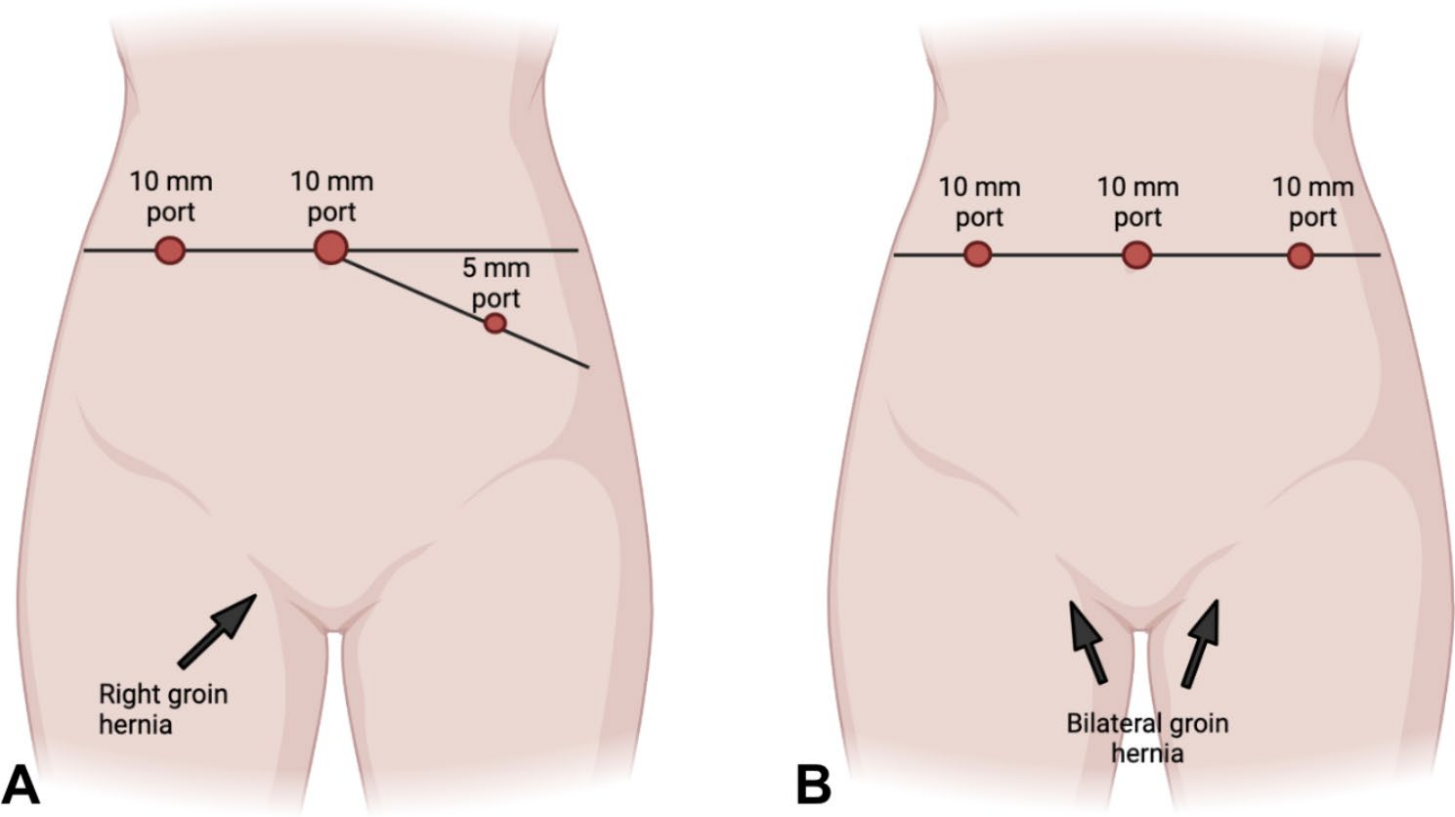
(**a**): Trocars placement for unilateral right hernia. (**b**): Trocars placement for bilateral hernia defects
  In bilateral hernias, however, the positioning changes (Fig. 2b). To operate in a comfortable and easy way, we suggest that the two trocars placed on the right and left sides (both 10 mm) should be inserted 1–2 cm above the transumbilical line, halfway between the midclavicular line and the anterior axillary line. We suggest that the trocar on the same side as the hernia defect should be 10 mm to allow for easy introduction and placement of the prosthesis. Consequently, in the presence of bilateral hernias, both of the operative trocars will be 10mm.
2. *Incision and detachment of the peritoneal wall (*Fig. 3a and b-4a*)*: An incision of 6–8 cm is made on the peritoneum, 4 cm above the deep inguinal ring, slightly to the side where epigastric vessels (*first landmark*) (Fig. 4b) can be observed in transparency along the line going from the anterior superior iliac spine (ASIS), to the medial umbilical fold (obliterated umbilical artery). It is crucial to avoid making a wide peritoneal incision as closing the peritoneum can be challenging, particularly at the end of the procedure when the surgeon may be fatigued. Additionally, a wider incision increases the risk of breakages and potential occlusive episodes based on adhesion caused by the mesh being exposed and the peritoneum protection being lost. This peritoneal incision allows carbon dioxide diffusion in the preperitoneal space carrying out a pneumo dissection. When creating the peritoneal flap, it is crucial to only separate the peritoneum, leaving the preperitoneal fat tissue in contact with the muscle. Dissecting through the preperitoneal fat can lead to potential injury, including vascular injuries in zone 1 or nerve injuries in zone 2.
  **Fig. 3:**
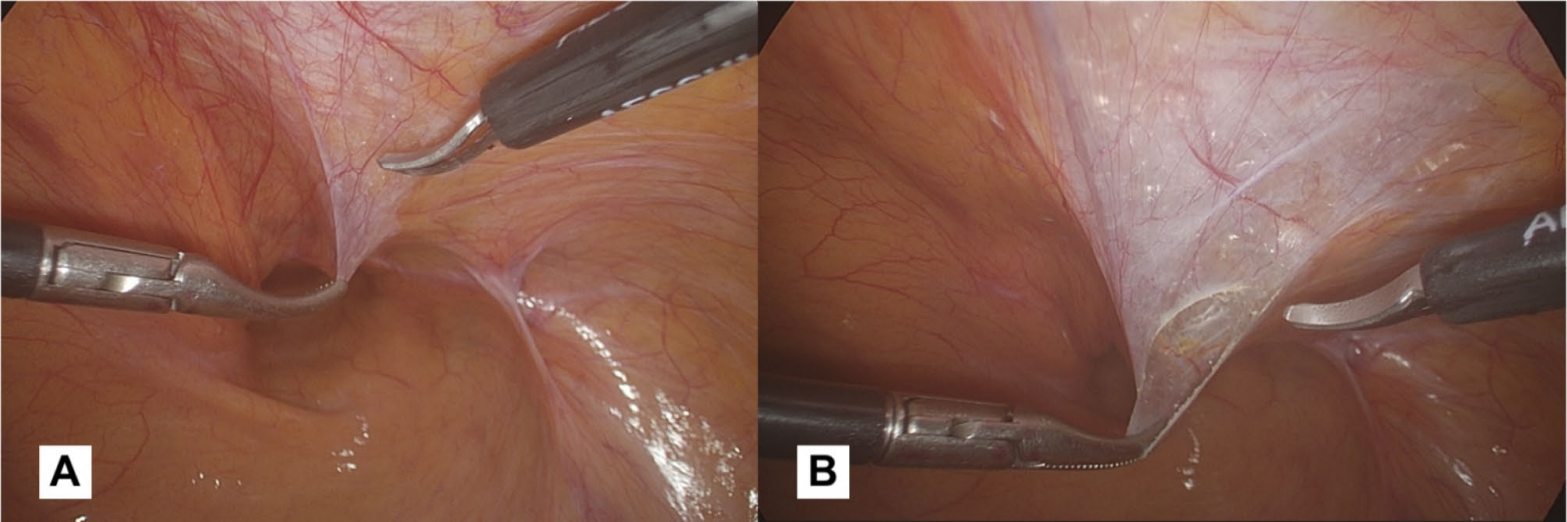
(**a**): Peritoneal flap opening 4 cm above the inguinal ring, slightly lateral to the pathway of epigastric vessels. (**b**): The peritoneal incision allows carbon dioxide diffusion in the preperitoneal space carrying out a pneumo dissection
  **Fig. 4:**
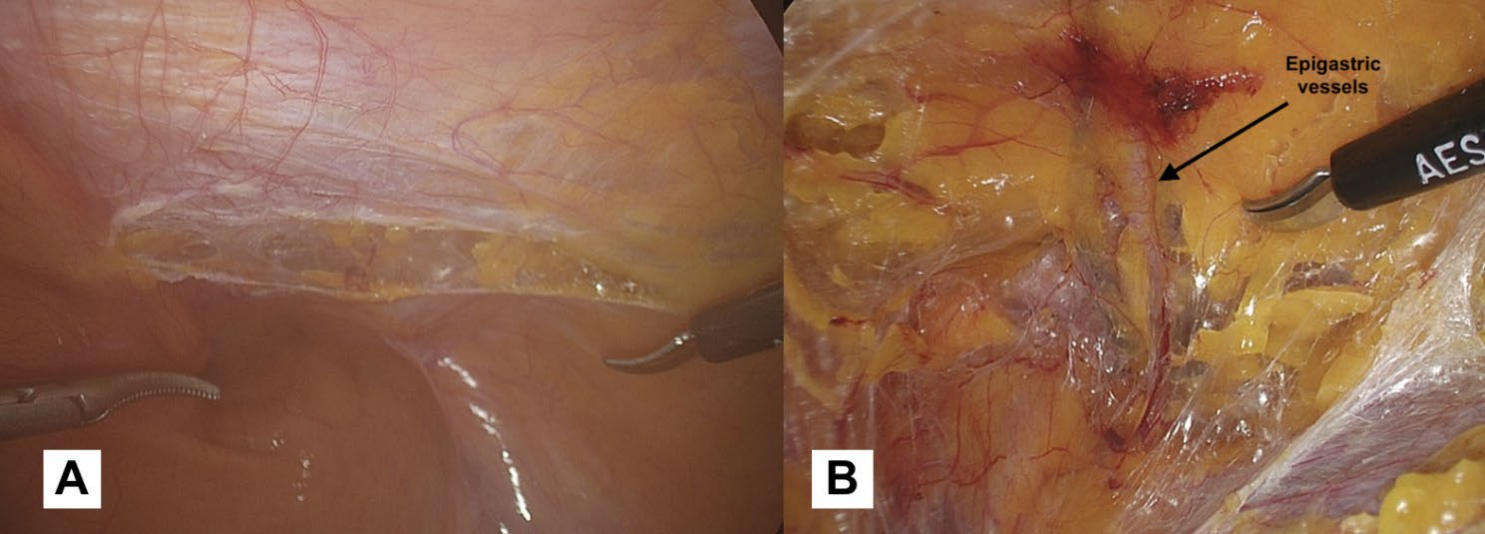
(**a**): We suggest for a peritoneal incision not exceeding 6–8 cm. (**b**): The epigastric vessels are the *first anatomical landmark*. It is advisable to identify these vessels before dissecting into zone 1
3. *Dissection of Cooper’s ligament (Zone 1) (*Fig. 5a and b*)*: if the hernia defect is direct, the time of hernia sac reduction will precede the search for the Cooper’s ligament, because the hernia defect will be located directly above the ligament itself.
  **Fig. 5:**
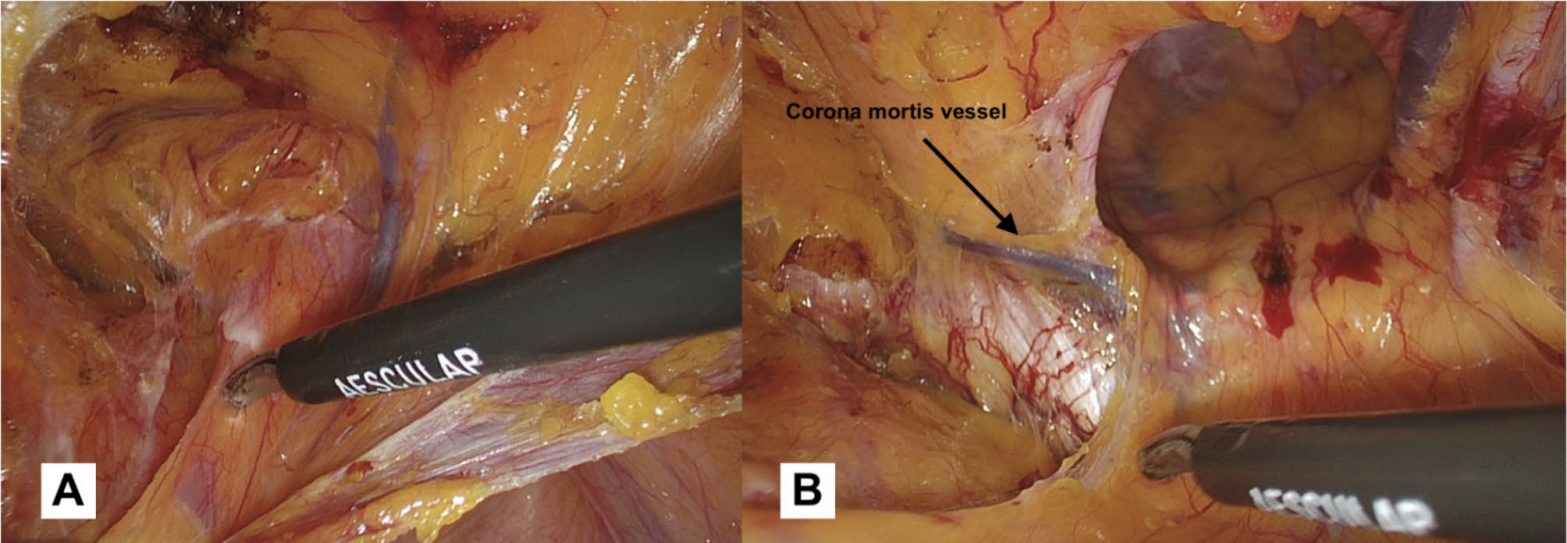
(**a**): Detachment of the direct hernia sac from the pseudosac. The direct defect is located superolaterally to the Cooper’s ligament. (**b**): Cooper’s ligament dissection: *the second anatomical landmark*. The vessel slightly above the ligament belongs to Corona mortis. In this area we suggest performing preventive coagulation
  However, the dissection begins in the medial part of the operating field with the aim of obtaining a clear view of the Cooper’s ligament (*second landmark*). During this phase, ensuring proper hemostasis is essential, particularly when working near the corona mortis. Based on our experience, we use bipolar forceps to perform preventative coagulation in this area to avoid dangerous bleeding. The vessels belonging to the corona mortis (an anastomosis between the obturator and the external iliac or inferior epigastric arteries or veins) are fragile and located in a deep position. If not pre-coagulated, they begin to bleed making the surgical field no longer clean, and when attempting to coagulate them with blind shots, these vessels tend to retract and disappear into the fatty tissue. When working in this area, it is always advisable to identify the femoral vein that runs laterally to the Cooper’s ligament. The preventive coagulation must be done keeping this vessel in mind and not approaching its course. We recommend coagulation that extends from the lateral (near the femoral vein) to the medial (towards the Cooper’s ligament). This reduces the risk of vascular injuries and ensures safe maneuvering.
  We agree with the indication given by Claus et al. [5]: the dissection in this phase should extend at least to the pubic symphysis and at least 2 cm below the pubic bone, within the Retzius space. This is useful for creating space for the prosthesis so that the necessary overlap is created with respect to any direct, femoral defects, and to ensure that the mesh is not lifted by the distended bladder. Also, it is crucial to be aware of the possible complications like vascular injuries, hematoma, bladder injuries, so we recommend to do a preventive coagulation of the vessels in zone 1, and to limit the dissection medially beyond the medial umbilical fold (obliterated umbilical artery), this will help to work outside of the bladder area. In the case of bilateral hernias, the dissection should be extended to the contralateral inguinal area through the Retzius space, until reaching the contralateral Cooper’s ligament.
4. *Dissection/reduction of the sac*: The surgical approach to a herniated sac differs significantly between direct and indirect hernias. In case of a direct hernia sac (Fig. 5a), it is necessary to hold the hernia sac and exert traction in a cranial direction. Counter-traction is applied by holding the pseudo-sac (transversalis fascia) and pushing it in a caudal direction. The weakened fascia transversalis is carefully separated and maintained in its original position. When repairing a direct hernia, the surgeon must take care to stay in the correct plane in order to prevent damage to the bladder, if it is included in the hernia [5].
  If the hernia is indirect (Fig. 6a-b), it is necessary to lift the sac, working with small bites and fine movements, and start dissecting following the profile of the hernial sac. At the beginning of this maneuver, it will be difficult to identify the profile of the sac from the tissue belonging to the spermatic cord. We recommend starting the dissection carefully in the superior part, in this way we should avoid the spermatic cord elements that usually run in the inferior part. In the case of a large indirect hernia, it is advisable to use a laparoscopic monopolar dissector and grasper. The dissector proves to be a versatile instrument, as it can be used for both holding the tissue and performing delicate dissection, reducing the need for frequent switching to monopolar scissors. For voluminous inguinal hernias, opening the sac can enhance the ability to follow its profile and facilitate a complete reduction. Another option is to open and abandon the sac, the choice of which - in our opinion - depends on the surgeon’s expertise. In particular during the ascending phase of the learning curve, this latter option may be easier, but it is associated with a higher risk of seroma formation.
  **Fig. 6:**
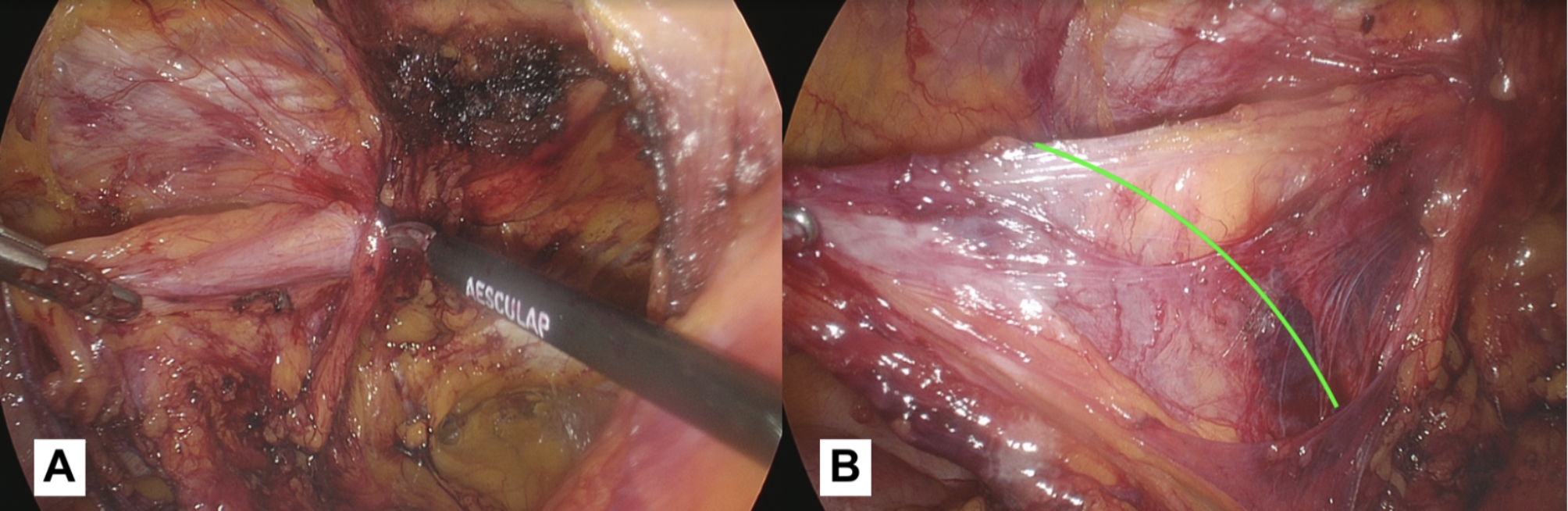
(**a**): Commencement of the hernia sac reduction procedure into the abdominal cavity. Due diligence must be exercised to carefully monitor the vas deferens course during this critical stage. (**b**): The hernia sac is almost completely reduced into the abdomen and the elements of the spermatic cord are completely isolated and detached from the hernia sac. It is possible to observe the cleavage plane (green line) between the sac and the elements that will lead to complete reduction
5. *Exploring for possible additional defects*: Although the hernia defect may appear to be evident, it is important to examine other regions to ensure that no additional hernia defects are present. In fact, it is important that the pathway external iliac vein is visible. Only in this way the femoral region can be explored for hernia defects. The femoral canal is located lateral to Cooper’s ligament and posterior to the iliopubic tract, a defect is located in the most medial part of the femoral triangle [8]. When an incarcerated femoral hernia defect is present, it is often necessary to enlarge the hernia ring through electrocoagulation, in that case we suggest using the monopolar hook.
  During this laparoscopic procedure, it is necessary to rule out the presence of obturator hernias. To do this, it will be necessary to explore the area inferolateral to the Cooper’s ligament, following the pathway of the obturator artery, in search of any obturator defects.
6. *Preparation of the peritoneal flap (Zone 3)*: the spermatic cord is preserved and detached from the peritoneum. The dissection is carried out following the vas deferens (*anatomical landmark 3 A*) until it crosses the external iliac vein in Zone 3. We suggest another way to determine if the parietalization step has been completed properly. Ideally, when lifting the peritoneal flap upward, the structures of the spermatic cord should be kept below and not lifted along with the flap (Fig. 7).
  **Fig. 7:**
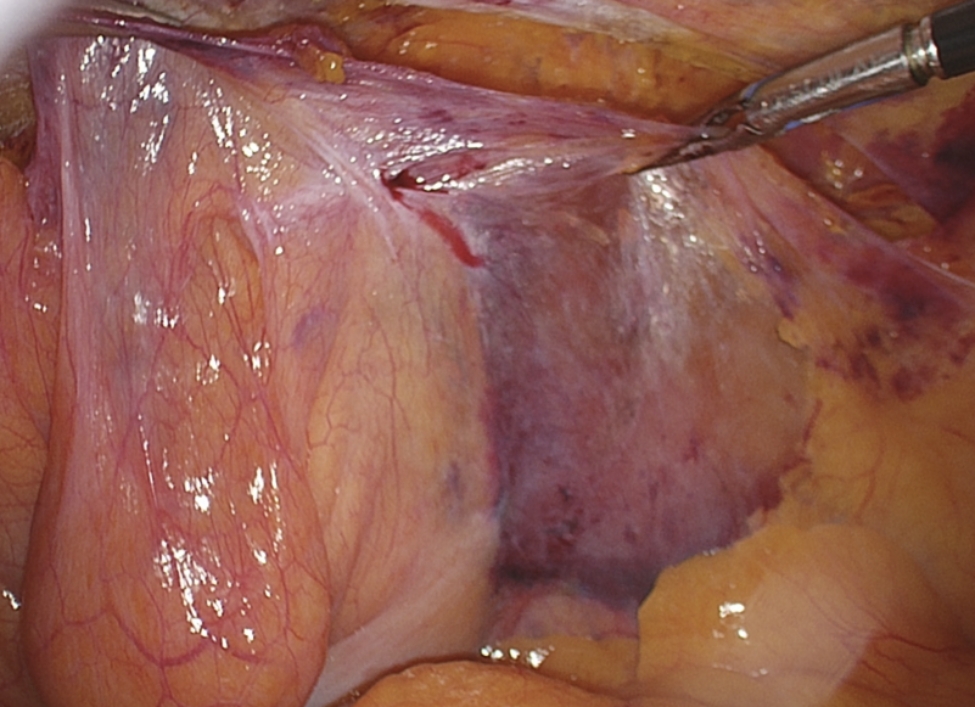
The result obtained after the complete parietalization of the vas deferens and the spermatic vessels
  In women undergoing TAPP, the round ligament of the uterus is a key anatomical consideration, but there is no consensus on whether it should be preserved or removed during surgery. The existing literature does not provide definitive guidance on this issue. The round ligament of the uterus is a structure that originates from the front part of the uterus and runs through the inguinal canal before terminating in the labia majora [12]. While the round ligament is not typically considered a key component of uterine suspension, it may play a role in maintaining the forward position of the uterine corpus [13]. Our recommendation is to make an effort in preserving the ligament during the surgical intervention, but this must not affect the correct positioning of the prosthesis.
7. *Direct defect closure (only in case of M3 defects according to European hernia society groin hernia classication) (*Fig. 8a-b*)*: the approach to suturing or plicating the transversalis fascia during the repair of a direct hernia is a matter of debate among surgeons. Some believe that suturing or plicating the fascia can help prevent the formation of a seroma. However, others question the necessity of this step and express concern that it may increase the risk of nerve injury. The closure of the fascial defect creates a uniform surface, providing a stable base for placement of the mesh. Failure to properly close a direct defect in the transversalis fascia can result in mesh migration, increasing the risk of recurrent herniation.We usually close the hernia defect using a continuous barbed suture with a synthetic absorbable monofilament (Polydioxanone) 2/0 Filbloc (Assut Europe). To ensure a successful and safe direct defect closure during laparoscopic groin hernia surgery, it’s important to keep the approach tension-free. This can be achieved by only working on the pseudosac during the plastic, the muscles should not be involved in the closure because of the risk of chronic pain and seroma. By doing so, the tension-free effect can be maintained. Additionally, it’s important to insert the needle superficially and mostly through the superior part of the defect to avoid any damage to the spermatic cord structures and to the iliohypogastric nerve that are close by.
  **Fig. 8:**
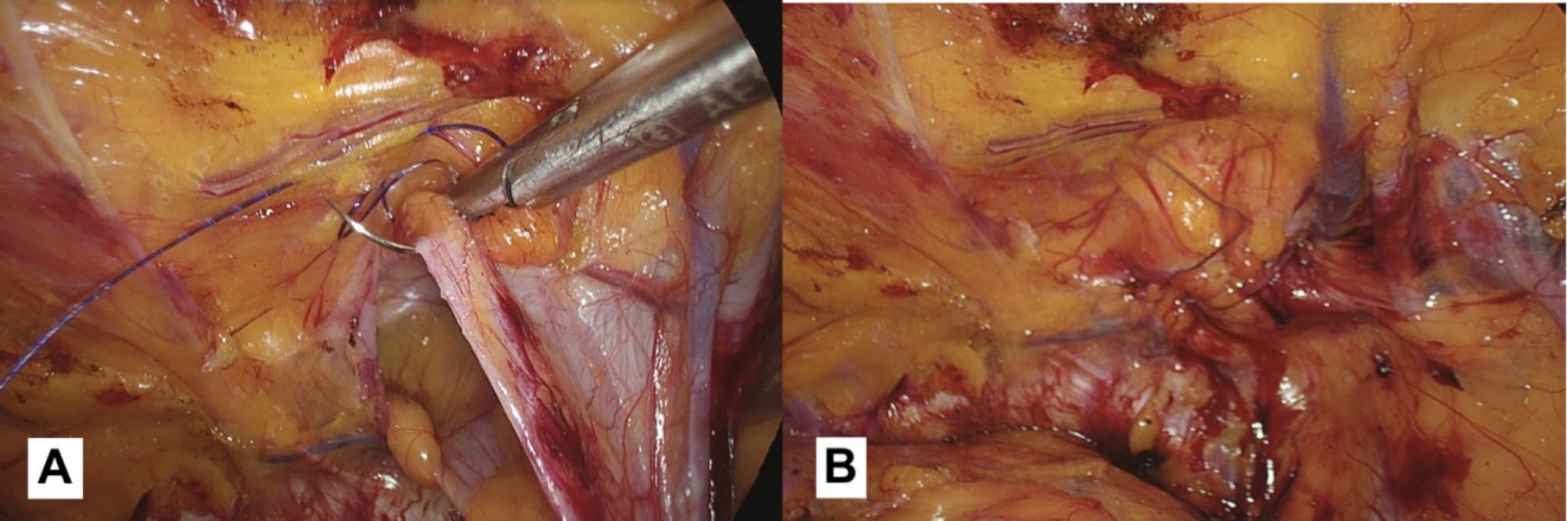
(**a**): In the case of large direct inguinal hernias, we recommend suturing the defect while paying attention to the elements of the spermatic cord that run below the defect. (**b**): The result obtained after the defect closure.
8. *Preparation of the mesh space*: Once any direct, oblique external hernia sacs have been reduced and the area has been explored for additional hernia defects, the search for the *anatomical landmark 3B* begins: *the spermatic vessels*. Once this landmark has been identified, work can begin on the lateral side of the surgical field to create space for the prosthesis. Before closing the peritoneum, using two graspers, the upper part of the peritoneum should be prepared and partially detached by pulling down and cephalad, this maneuver makes the peritoneum more compliant and, consequently, the closure easier.
9. *Placement of the prosthesis (*Fig. 9a*)*: our group uses a a 10.3 cm x 15.7 cm polypropylene, large pore, 3D mesh (3DMax™ Mesh, BD, 100 Crossings BoulevardWarwick, Rhode Island 02886, United States): a curved, three-dimensional polypropylene prosthesis that is pre-shaped. Due to the curvature, there is a right and left prosthesis. Our standard size is large, but in specific cases, an extra-large (12.2 cm x 17.0 cm) has been utilized (19/487, 3.9%). In fact, for large direct hernias where the fascial defect is left unclosed, the use of extra-large prosthetic mesh is advised to ensure adequate overlap and coverage of the hernia defect. This is critical in reducing the risk of recurrent herniation and ensuring a successful outcome of the surgical repair. The prosthesis is grasped at the medial portion, introduced through the 10 mm trocar, positioned in correspondence with the medial part of the defect and held in place from that side. Meanwhile, a grasper introduced through the 5 mm trocar is used to smooth out any defects and position the mesh in contact with the posterior inguinal wall. To prevent recurrences, it is important that the prosthesis creates an overlap of 3–4 cm relative to the margin of hernia defects. This ensures that the prosthesis covers the entire area of the defect and helps to prevent the hernia from recurring in the future. In the case of bilateral hernias, two meshes will be used that will overlap by 2–3 cm at the prevesical space of Retzius. Overall, proper surgical technique and the use of appropriate materials are key factors in achieving successful laparoscopic hernia repair outcomes. For several years now, we haven’t fixed the prosthesis in any way. In our view, when using this surgical technique and type of prosthesis, there is no requirement to secure the mesh. Our hernia recurrence rate (2/487, 0.41%) is not superior to that reported in the article by Lovisetto et al. (0.6%) [14], which relates to our previous experience with a different prosthesis and mesh fixation using metal clips, and is on par with the recurrence rates found in the literature (median of 2.3%) [1]. However, when using a different type of prosthesis, mesh fixation may be necessary. We strongly advise against the use of clips, as they seem to have a higher rate of both short and long-term chronic pain. As an alternative, we recommend using fibrin glue which seems to be associated with lower postoperative pain rates [4]. Anyway, we definitely agree with Claus et al.: fixing the mesh does not prevent problems arising from earlier stages of the operation that were not performed properly [5].
  **Fig. 9:**
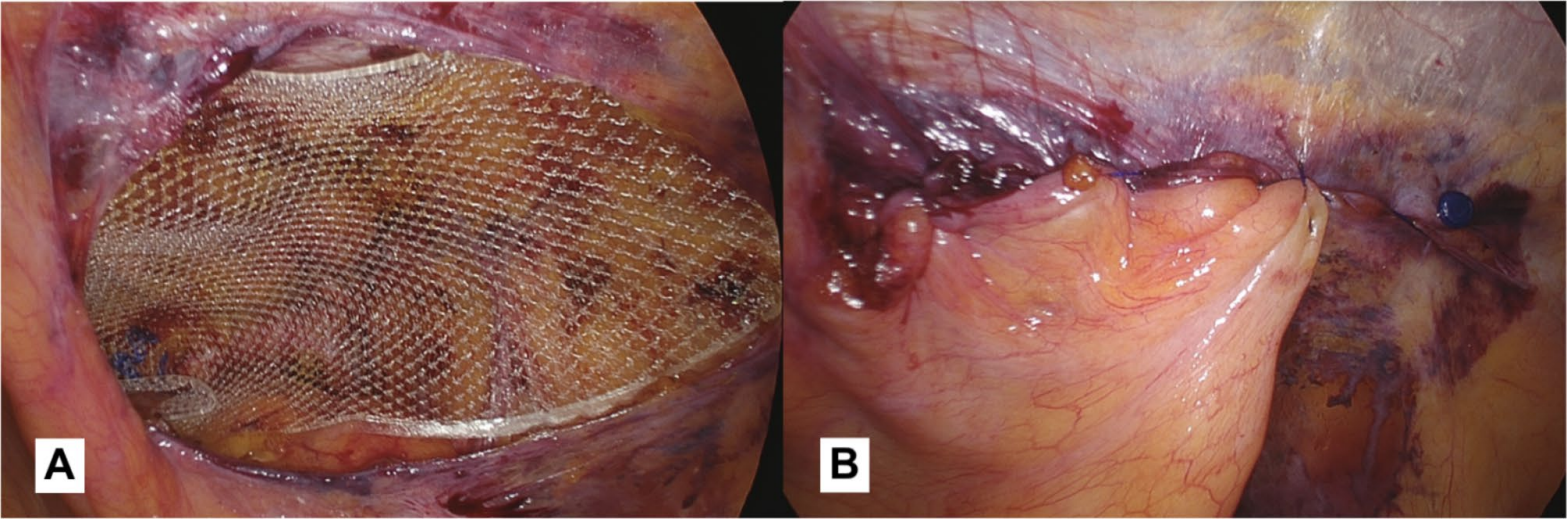
(**a**): Polypropylene 3D mesh placed: a curved, three-dimensional, pre-shaped prosthesis. (**b**): The image shows the closed peritoneum. We suggest using the redundant direct hernia sac, when present, to reinforce the suture
  Several years ago, some anatomical studies in literature that suggested shifting the upper boundary of the pain triangle 2 cm above the iliopubic tract, as the nerve pathways in this area can be not always constant, in fact the nerve branches of the lateral cutaneous nerve of the thigh, the femoral branch of the genitofemoral nerve and the femoral nerve can extend up to 2 cm above the iliopubic tract [15]. Building on this idea and based on our experience, we propose that there could be an additional sixth triangle - a second pain triangle, positioned above the first one, above the inguinal ligament, and to the lateral side of the triangle that identifies the region of indirect hernias (Fig. 1a). The proposal for the addition of a second pain triangle comes from our observations of patients in whom the mesh was fixed using metal clips. We found a higher rate of post-operative pain in both the short and long term, in patients where clips were placed within this area. This led us to believe that not only the region where nerves are supposed to run is involved in such complications, but also the transversus abdominis muscle region above. This is most likely due to the varying anatomy of nerve branches, which may extend over the ileopubic tract [14], [16]. Additionally, we believe that, due to anatomical reasons, the metal clip penetrating the and injuring the endomysium of the muscle fibers, endowed with sensitive nerve endings, can lead to chronic pain. Hence, we suggest adding an extra anatomical landmark to indicate the hazards of securing clips in this region potentially causing chronic pain.
10. *Closure of the peritoneal flap (*Fig. 9b*)*: Eventually, the mesh is covered with the peritoneal flap, closed by a continuous barbed monofilament 2 − 0 suture (Filbloc, Assut Europe). We recommend suturing starting from the right side of the flap, as this allows the surgeons to tackle the most uncomfortable part first and then move on to the easier part. This approach allows the surgeon to work in a comfortable and efficient manner, which can help to reduce the risk of complications and improve the overall outcome of the surgery. The redundant sac, if present, will be included in this suture and used such as a patch. Another tip we recommend is to cut the thread as close as possible to the peritoneal wall. This will help to avoid entrapment of the intestinal loops with subsequent intestinal occlusion due to adhesions, which can be caused by leaving the free margin of the barbed suture too long [14], [17].
  Finally, we recommend for those who approach this type of surgery for the first time, to commence their experience by starting with small direct inguinal hernias located on the left. In this way, the approach will be gradual and lighter, as the trocar of the dominant hand will be farther away from the hernia and it will be easier to make fine and measured movements.

## Results

The technique presented was performed on 362 patients who underwent TAPP procedure from March 17, 2015 to October 7, 2022: 332 men and 30 women (91.7% vs. 8.3%) with a median age of 62 years old (IQR 51–71) (Table 1). 487 hernia defects were repaired: 277 indirect hernias (203 unilateral and 37 bilateral); 175 direct hernias (101 unilateral and 37 bilateral); 34 femoral hernias (28 unilateral and 3 bilateral) and one unilateral obturator hernia (Table 1).

**Table 1:** Table describing the types of inguinal hernias repaired during our experience

HERNIA REPAIR		N.	%
INDIRECT^a^	MONOLAT.	203	41,7%
	BILAT.	37	7,6%
TOT		277	
DIRECT^a^	MONOLAT.	101	20,7%
	BILAT.	37	7,6%
TOT		175	
FEMORAL^a^	MONOLAT.	28	5,7%
	BILAT.	3	0,6%
TOT		34	
OBTURATOR^a^	MONOLAT.	1	0,2%
	BILAT.		
TOT		1	
**TOT. DIFETTI RIPARATI**	**487**		

Abbreviations: TOT (total); MONOLAT (monolateral); BILAT. (bilateral)

Categorical variables (a) are expressed in frequency (%).

The median duration of the surgery was 55 min (IQR 40–70) (Table 2). 14 repairs were done in an emergency setting, 348 were elective (3.9% vs. 96.1%). We usually use a size L prosthesis, a XL size prosthesis was used in 19 cases (19/343, 5.2% vs. 94.8%).

**Table 2:** The table describes the general characteristics of patients and the rate of emergency repairs performed

		N.	%	Median (IQR 25–75)
GENDER^a^	M	332	91,70%	
	F	30	8,30%	
AGE^b^				62 (51–71)
EMERGENCY^a^	NO	348	96,13%	
	YES	14	3,87%	
**TOT**		362	100%	

Abbreviations: TOT (total)

Categorical variables (a) are expressed in frequency (%) and continuous variables (b) are expressed as median (IQR)

Direct defect suturing was performed 15 times (15/347; 4.1% vs. 95.9%) (Table 3).

**Table 3:** Table describing the median duration of surgery, type of prosthesis used, and frequency of direct defect closure

		N.	%	Median (IQR 25–75)
XL MESH^a^	NO	468	96,10%	
	YES	19	3,90%	
DEFECT CLOSURE^a^	NO	472	96,92%	
	YES	15	3,08%	
DURATION (min)^b^				55 (40–70)

Categorical variables (a) are expressed in frequency (%) and continuous variables (b) are expressed as median (IQR)

The median follow-up time is 28 (IQR 15–44) (Table 4).

**Table 4:** Table describing the rate of complications and recurrence during our experience

		N.	%	Median (IQR 25–75)
COMPLICATIONS (without recurrence)^a^	NO	485	99,59%	
	SI	2	0,41%	
RECURRENCE^a^	NO	485	99,59%	
	SI	2	0,41%	
FU DURATION (months)^b^				28 (15–44)

Abbreviations: FU (follow-up)

Categorical variables (a) are expressed in frequency (%) and continuous variables (b) are expressed as median (IQR)

Regarding complications, we recorded two relapses (after 12 and 16 months from surgery) treated with an anterior approach using the Lichtenstein technique with a hernia mesh, a seroma regressed with conservative treatment, one episode of inguinal neuropathic pain (VAS = 8) lasting for 18 months and then self-limited and an episode of occlusion based on adhesion caused by the tail of the peritoneal flap suture. The patient underwent surgery with success and recovered without sequelae.

## Discussion

Laparoscopic surgery has become the preferred technique for inguinocrural hernia repair in selected cases such as bilateral hernias and recurrences after open anterior approach due to its well-established advantages. These advantages include lower rates of postoperative pain, a faster return to normal activities, a lower incidence of infections, similar recurrence rates, and less chronic pain [1], [18]. Several studies have shown that laparoscopic hernia repair has a longer learning curve, and the surgeon performing the procedure must be well-trained in laparoscopic surgery [19–21]. Becoming an experienced surgeon in laparoscopic hernioplasty requires achieving a late recurrence rate of less than 1% [1].

Several studies have evaluated the learning curve for laparoscopic hernia repair using different parameters such as operation time [22–24], conversion rate [22], and number of recurrences [22], [25]. The findings of these studies suggest that it may take between 20 and 240 procedures for the learning curve to plateau and reach a stable level that is comparable to that of experienced surgeons.

During this learning curve period, operation time, morbidity, and recurrence rates tend to decrease as the trainee gains experience and proficiency in the operative technique.

Our study, consistent with the findings of Bökeler et al. [24], has demonstrated a recurrence rate of less than 1% for patients undergoing TAPP hernia repair by trainees from the outset, which is a significant finding. Additionally, our results obtained from the surgeons enrolled in the hernia program reveal that the only parameter exhibiting a notable learning curve is the operation time. This indicates that with a well-designed educational program and rigorous standardization of the operative approach, trainees can achieve exceptional outcomes and low recurrence rates right from the beginning of their training.

Two key factors for successful laparoscopic hernia repair are the standardization of the technique and the number of procedures performed by the surgeon [18]. In our center, with over 30 years of experience in laparoscopic hernia repair and more than 3500 procedures executed, standardization was achieved by having only two surgeons perform TAPP between 1992 and 1994. However, their recurrence and complication rates were higher than those obtained after 1994 when the technique was standardized. Over the last seven years, two young surgeons in training have executed 168 surgeries with a median operation time of 93 min, specifically 83 min for unilateral hernias and 115 min for bilateral hernias. The durations of the first fifty mentored procedures significantly contribute to the number mentioned above. However, based on our data, we can affirm that from the seventieth individual procedure onwards, the durations fall within the confidence interval of an experienced surgeon and start approaching a plateau. We hypothesize that this plateau will be reached around the hundredth procedure. Importantly, there has been no increase in the recurrence rate during this time period. This highlights the success of the standardized approach and the importance of gradually building surgical expertise through the execution of numerous procedures.

Multiple studies have recorded the progress in the learning curve using operative time as a variable. There is no consensus about the number of procedures required, it ranges from 20 to 240 [19–21].

We have developed a comprehensive learning program for laparoscopic hernia repair that follows a step-by-step approach. The training begins by having the surgeon in training perform step 10, which involves closing the peritoneum at least ten times while the operative time is recorded. We consider peritoneal suturing to be one of the most challenging steps in the procedure, as it occurs at the end of the operation, the operator’s position is not ideal, and the surgical field is vertical, not horizontal. Despite these difficulties, this step is critical for preventing serious complications [14], [17].

In phase two of the program, the surgeon performs steps 9 and 10 while the operative time is recorded. Subsequently, the surgeon performs the entire procedure under the guidance of an expert operator holding the camera. During this phase, we recommend that the surgeon repair at least 50 easy defects, starting from direct, left, and M1 or M2 according to European hernia society groin hernia classification [22], before advancing to the next phase. In the next phase, the operator must repair 50 defects of progressively increasing difficulty, such as bilateral defects, but not recurrent defects. Finally, after successfully repairing 100 defects, the surgeon should be able to perform the procedure independently with proficiency.

In the literature, we found another study that divided the procedure into three phases similar to ours: closure of the peritoneum, placement of the prosthesis, and dissection of the surgical field. This study states that the learning curve for peritoneal closure is reached after 22 cases, for prosthesis placement after 32 cases, and for dissection after 75 cases [23].

This standardized training approach enables the surgeon in training to gradually acquire the necessary skills and expertise to perform laparoscopic hernia repair with confidence and autonomy. The inclusion of a progressive learning pathway allows for a more effective and efficient training process, reducing the risk of complications and improving patient outcomes.

As stated by Bökeler et al. [20] the inclusion of laparoscopic hernia repair as a fundamental component of the trainee program is imperative. However, this should be done under certain preconditions. These include the establishment of TAPP in the clinical setting, strict standardization of the operative technique, and a well-structured educational program.

It is important to note that a learning curve does not necessarily lead to higher complication and recurrence rates, provided the aforementioned preconditions are met.

An important role in reaching the plateau of the learning curve faster could be played by simulators. In literature, there are various systems, both virtual and anatomical, that allow for the reproduction of the procedure. There have been some criticisms, particularly regarding virtual simulators, as they do not provide tactile feedback and differ significantly from real intraoperative situations [24]. Anatomical simulators, on the other hand, require substantial resources to be set up, whether in terms of finances or time, which may not always be feasible for professionals who work extensively. In our experience, the residency program includes regular attendance and various practical examinations at a laparoscopic simulation center called Niguarda Laparoscopic Arena, which, in our opinion, plays a role in facilitating trainees in reaching the plateau of the learning curve.

## Conclusion

The widespread adoption of laparoscopic surgery for groin hernia repair has been hindered by the need for advanced laparoscopic skills and familiarity with the anatomy of the posterior inguinal region. In this study, a standardized and reproducible laparoscopic TAPP technique was presented. The introduction of the new anatomical landmark and the modified numbering system provide a more comprehensive standardization of the surgical technique. The findings demonstrate the feasibility and reliability of the laparoscopic TAPP technique and serve as a useful resource for surgeons who are approaching this surgical technique for the first time.

## Data Availability

All data is contained within the manuscript. The datasets used and analyzed during the current study available from the corresponding author on reasonable request.
